# Bevacizumab plus preoperative chemotherapy in operable HER2 negative breast cancer: biomarkers and pathologic response

**DOI:** 10.1007/s12094-013-1006-4

**Published:** 2013-02-09

**Authors:** P. Sánchez-Rovira, M. A. Seguí, A. Llombart, E. Aranda, A. Antón, A. Sánchez, M. Lomas, A. Jaén, M. Fernández, I. Porras, E. Dalmau, S. Morales, J. de la Haba-Rodríguez

**Affiliations:** 1 Servicio de Oncología Médica, Complejo Hospitalario de Jaén, Avda. del Ejército Español, 10, 23007 Jaén, Spain; 2 Medical Oncology, Corporació Sanitària Parc Taulí, Barcelona, Spain; 3 Medical Oncology, Hospital Universitàri Arnau de Vilanova, Lleida, Spain; 4 Medical Oncology, Hospital Reina Sofía, Córdoba, Spain; 5 Medical Oncology, Hospital Universitario Miguel Servet, Zaragoza, Spain; 6 Medical Oncology, Hospital Universitario Virgen de la Victoria, Málaga, Spain

**Keywords:** Bevacizumab, Biomarkers, Breast cancer, Combined modality therapy, Neoadjuvant therapy

## Abstract

**Purpose:**

The primary aim of this trial was to assess the rate of pathologic complete responses (pCR) of doxorubicin/cyclophosphamide (AC) followed by bevacizumab/docetaxel (BT), as neoadjuvant therapy for breast cancer (BC). Furthermore, the association between biomarkers and the pCR was explored.

**Methods:**

Patients with HER-negative operable stage II–III BC ≥2 cm were enrolled. Four cycles of AC (A 60 mg/m^2^ and C 600 mg/m^2^, every 3 weeks) followed by 4 cycles of BT (B 15 mg/kg and T 75 mg/m^2^, every 3 weeks), were planned. A core-biopsy was performed for biological markers assessment.

**Results:**

Seventy-two women were included. Forty-three (63 %) patients were hormone receptor-positive. Sixty-four (89 %) completed the planned treatment, and 66 evaluable patients underwent surgery (92 %): a pCR was achieved in 16 of them (24, 95 % CI 15–36 %). pCR was significantly higher in tumors hormone receptor-negative, and in those with Angiotensin II type 1 receptor (AGTR1) protein overexpression. The overall clinical response rate was 86 % (95 % CI 76–93 %), including 42 complete responses. No unexpected toxicities or treatment-related deaths were observed.

**Conclusion:**

This regimen showed a remarkable clinical and pathological activity: the suggested relation between pCR and AGTR1 overexpression should be confirmed in larger trials.

## Introduction

It has been suggested that the pathological response of the tumor and the number of affected lymph nodes are the most important prognostic factors in terms of prolonging survival after neoadjuvant chemotherapy [1, 2].

The combination of docetaxel with anthracyclines represents one of the standards in the primary treatment of breast cancer (BC) [3].

Numerous studies have demonstrated that therapeutic disruption of nascent vasculature is effective in mediating tumor regression [4]. The anti-angiogenic agent bevacizumab (Avastin^®^; Genentech Inc., San Francisco, CA, USA; Hoffmann-La Roche Ltd, Basel, Switzerland) is a humanized monoclonal antibody with binding specificity for vascular endothelial growth factor (VEGF): it has the potential to decrease the size of a tumor by reducing its blood supply and enhancing the activity of cytotoxic therapies.

The addition of a drug with a biological mode of action, such as bevacizumab, to a docetaxel-based cytotoxic regimen may increase the antitumor activity and prognosis of women with BC without increasing the treatment toxicity. Indeed, there is evidence from in vitro studies suggesting that docetaxel has antiangiogenic effects which increase synergistically with bevacizumab administration [5].

Neoadjuvant therapy has been used to assess the relevance of biological markers as potential predictive factors for efficacy. C-erbB2, p53, Ki-67 labeling index, VEGFR, and hormone receptor (HR) status, among others, has been evaluated as predictive markers in several trials, with contradictory or inconclusive results [6–14]. Therefore, this issue remains unsolved, being of special relevance in antiangiogenic combination treatments.

The present multicenter, open-label, phase II study was designed to evaluate the efficacy and the safety profile of a sequential treatment with the classical regimen of doxorubicin/cyclophosphamide (AC) followed by the combination of bevacizumab plus docetaxel (BT) in the neoadjuvant treatment of operable BC, as well as the possible correlation between the expression of certain biomarkers and the efficacy of this therapeutic approach.

## Patients and methods

### Eligibility criteria

The primary objective was to assess the efficacy, measured as the rate of pathologic complete responses (pCR) at surgery after receiving sequential induction chemotherapy with AC followed by BT. Secondary objectives included the assessment of the objective response rate (ORR) and safety profile of the regimen, the percentage of breast-conserving surgery, and the study of the potential association between several recognized biomarkers in the baseline biopsy and the pathological response to the study treatment.

The study was performed after approval by the Independent Ethics Committee of each site and in accordance with the Declaration of Helsinki, the Good Clinical Practices, and local ethical and legal requirements. Before study entry all patients provided written informed consent according to local ethical committee regulations. A specific informed consent was asked to patients to provide a tumor sample to be analyzed for biomarkers: it was not mandatory to accept this proposal to be included in the study.

Patients with histological or cytological confirmed operable stage II–III BC ≥2 cm, HER2-negative, without prior treatment for BC, were enrolled at 6 sites in Spain. Other inclusion criteria included: Eastern Cooperative Oncology Group (ECOG) performance status (PS) ≤1, adequate hepatic, renal, and bone marrow function, and a left ventricular ejection fraction (LVEF) ≥55 %. Patients were excluded from participation if they had other comorbidity conditions including neuropathy >grade 2 according to National Cancer Institute-Common Terminology Criteria for Adverse Events (NCI-CTCAE), and clinically significant cardiac disease (New York Heart Association, Class ≥II). In addition, patients with a prior history of bleeding or coagulopathy with risk of bleeding, and current use of full dose or parenteral anticoagulants or chronic daily treatment with aspirin (<325 mg/day) also were excluded.

### Treatment schedule

Patients should receive four cycles of AC regimen (doxorubicin at 60 mg/m^2^ iv bolus over 15 min plus cyclophosphamide at 600 mg/m^2^ iv infusion over 5–60 min at day 1 every 3 weeks) followed by four cycles of BT regimen (bevacizumab at 15 mg/kg iv infusion followed by docetaxel at 75 mg/m^2^/day iv at day 1 every 3 weeks).

Patients were scheduled to undergo surgery at least 4 weeks after receiving the last bevacizumab and/or docetaxel dose. Breast-conserving surgery was done whenever possible (diameter <2 cm, absence of central localization and disseminated calcifications).

### Study assessments

Before study entry, all patients were evaluated for their axillary nodes and breast disease, including a complete anamnesis and physical examination, a radiological examination using mammography, ultrasonography and/or magnetic resonance imaging (MRI) of the breast, a routine blood analysis (hematology and biochemistry), an assessment of the ECOG PS and a measurement of the LVEF. In addition, chest radiography and computed tomography (CT) of the chest and abdomen, as well as bone scintigraphy were performed within 28 days before the start of the study treatment to exclude the presence of metastases. Performance status and vital sign assessments, routine blood analysis and urinalysis were repeated before each treatment cycle until final follow-up visit, which occurred 12 months after treatment. LVEF was repeated before first BT infusion and after surgery.

Patients underwent a core-biopsy of the primary tumor for the diagnosis and the biological characterization of the tumor, including HR and HER-2 status in all patients, and Ki-67 labeling index (low if <15 % and high if ≥15 %), and other biological markers (VEGF, VEGFR, PAKT, PMAPK, KISS1, RKISS1, HIF, eNOS, AGTR1, and IGF) in those who give their consent to have the biomarkers analyzed. The protocol for collection and measurement of these markers has been described previously [10].

Tumor evaluation was indicated after each cycle by physical exploration, and every 4 cycles (after AC regimen, and then after completion of BT cycles) by breast MRI and/or ultrasonography. RECIST v.1.0 response guidelines were used [15] to define tumor responses: imaging-based evaluation was always being done rather than physical examination unless the lesion(s) being followed could not be imaged but was assessable by clinical exam.

An anatomopathological evaluation of the surgical sample was done after surgery, as well as an assessment of the same biological markers obtained at the basal core-biopsy. An absence of invasive tumor cells within the breast and lymph nodes was classified as a pCR, according to the Miller and Payne criteria [16].

Toxicity was evaluated at the initiation of each cycle and until 28 days after the last study drug dose administration, using the NCI-CTCAE version 3.0.

### Statistical considerations

A Simon two-stage design [17] was used to calculate the required sample size, based on testing a null hypothesis H_0_ pCR rate ≤20 % and an alternative hypothesis H_1_ pCR rate ≥35 %, with an alpha level of 0.05 and a statistical power of 80 % (beta error = 0.20). Seventy-two evaluable patients were required to achieve these goals. The study would be stopped if ≤5 pCR resulted after evaluation of 22 patients.

The primary efficacy analysis for clinical response was performed on the intention to treat (ITT) population, defined as all included patients. Population assessable to clinical response included patients with no major protocol violations, who had received at least 2 cycles of treatment (those with early progression after first cycle were also included) and with a tumor evaluation after those 2 cycles. To be assessable to pathological response, patients should have received at least 2 cycles of treatment, and have had their tumor anatomopathologically analyzed, without major protocol violations. Safety population comprised all included patients who received at least one administration of any study drug.

Frequency and percentage of pCR and ORR, together with the IC 95 %, were presented. The association between baseline characteristics, including biomarkers, and pathological/clinical response was evaluated by logistic regression in both univariate and multivariate analyses. All *p* values were two-sided with a significance level of 0.05. The statistical analyses were run using SAS version 9.2.

The guidelines for the reporting of tumor marker studies (REMARK) [18] were followed to analyze and present data on studied biomarkers.

## Results

### Patient characteristics

**Fig. 1:**
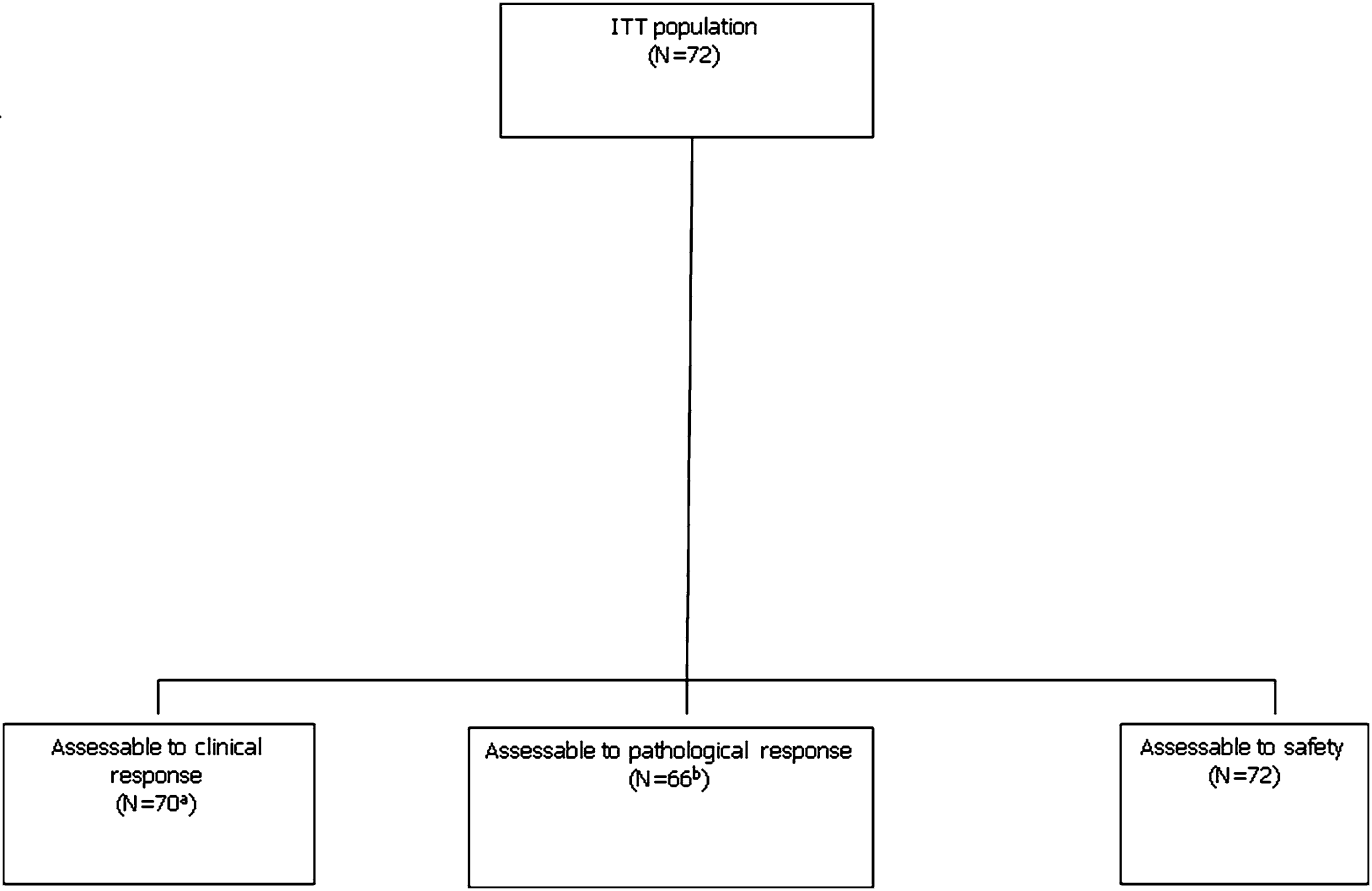
Disposition of patients. **a** Two patients were excluded because of major protocol violation (HER2 3+). **b** One patient was excluded because of major protocol violation (HER2 3+)

A total of seventy-two women from six Spanish centers were enrolled from December 2007 to March 2009. Figure 1 details patient disposition and Table 1 presents the basal characteristics of the patients. All but one woman had an ECOG PS of 0. Median tumor size (physical examination) was 5 cm (range 2–15). Forty-three patients (60 %) had tumors that were estrogen (ER) and progesterone receptor (PgR) positive.

**Table 1:** Patient characteristics

	No. of patients	%
Age (years)		
Median	46	
Range	24–73	
Menopausal status		
Premenopause	52	72
Menopause	20	28
Hormone receptor status		
ER+/PgR+	43	60
ER+/PgR−	12	17
ER−/PgR+	3	4
ER−/PgR−	14	19
Histologic type		
Infiltrating ductal carcinoma	69	96
Infiltrating lobular carcinoma	2	3
Metaplasic carcinoma	1	1
T classification		
T1	2	3
T2	42	58
T3	23	32
T4	5	7
Clinical anatomic stage		
IIA	14	19
IIB	32	45
IIIA	17	24
IIIB	6	8
IIIC	3	4

*ER* estrogen receptor, *PgR* progesterone receptor

### Treatment compliance

Sixty-four patients (89 %) completed the planned treatment (4 cycles of AC → 4 cycles of BT → surgery). AC was administered for four courses in 70 patients, and discontinued after the second cycle because of progression in one patient, and after the third cycle in another due to major protocol deviation (HER2-positive). BT was not administered to 2 patients because of consent withdrawal; four patients did not complete the planned 4 cycles of BT because of hypersensitivity reactions to docetaxel (1 patient after the first BT cycle, 2 after the second cycle, and 1 after the third cycle); one patient did not receive bevacizumab on cycles 3 and 4 due to uncontrolled hypertension.

A total of 286 cycles of AC were delivered. Administration was delayed in 53 cycles (37 patients), mostly (64 % of delays) due to causes not related to the treatment (organizational problems at the study center such as holidays, agenda adjustments or delay in the results of diagnostic tests): in 30 % of cases the delay was due to hematologic toxicity. The dose of doxorubicin was reduced in 4 cycles, due to hematologic toxicity, and the dose of cyclophosphamide in 1 cycle (non-hematologic toxicity). Bevacizumab + docetaxel was administered to 68 patients (264 cycles), and 46 cycles (35 patients) were delayed, in 78 % of them due to causes not related to the treatment (organizational problems at the study center), and in 15 % due to hematologic toxicity. Dose of docetaxel was reduced in 15 cycles (12 patients), mainly due to non-hematologic toxicity (10 cycles). Per protocol, there were no adjustments in the dose of bevacizumab.

### Safety

All patients were evaluable for safety. There were no surgical complications. The maximum toxicity per patient grade 3/4, irrespective of relationship to study treatment, is summarized in Table 2. Eleven patients presented 13 severe AEs classified as probably related to any study medication: 6 cases of febrile neutropenia (4 patients grade 3 and 2 patients grade 4), 4 of neutropenia (1 patient grade 3 and 3 patients grade 4), 2 of grade 3 mucositis, and 1 case of grade 3 vomiting. Cardiac dysfunction with heart failure symptoms was not observed. No patient died of treatment toxicity.

**Table 2:** Maximum toxicity (any grade 3/4 toxicity) per patient according to NCI-CTC criteria v3.0 (*N* = 72)

	Grade 1/2	Grade 1/2 (%)	Grade 3/4	Grade 3/4 (%)
Hematological toxicity				
Neutropenia	7	10	8	11
Febrile neutropenia^a^	1	1	8	11
Non-hematological toxicity				
Mucositis	31	43	14	19
Nausea	36	50	7	10
Vomiting	20	28	5	7
Fatigue	39	54	5	7
Allergic reaction/hypersensitivity	10	14	4	6
Diarrhea	15	21	2	3
Alopecia	35	49	1	1
Pain	35	49	1	1
Fever (without neutropenia)	13	18	1	1
Nail changes	11	15	1	1
Hypertension	6	8	1	1

^a^Febrile neutropenia: fever of unknown origin without clinically or microbiologically documented infection (Absolute neutrophil count <1.0 × 10^9^/L, fever ≥38.5 °C)

### Efficacy

Sixty-seven patients underwent surgery, being performed breast-conserving surgery in 42 of them (63, 95 % CI 50–74).

Four patients did not undergo surgery due to consent withdrawal (2 patients), disease progression (1 patient), and toxicity (1 patient). One patient was excluded from the pathological response assessable population due to major protocol deviation (HER2-positive). A total of 16/66 patients (24, 95 % CI 15–36 %) achieved pCR. No association was observed between pCR and age or tumor size (≤2 vs. >2 cm). A statistically significant association (*p* = 0.0094) was founded between the rate of pCR and the HR status (pCR rate of 54 and 17 % in HR− and HR+ patients, respectively) (Table 3).

**Table 3:** Pathological response rate according to biological markers analysis

Biological marker	Analyzed cases	pCR *N* (%)	*p*-value
Hormonal receptor			*0.0094*
−	13	7 (54)	
+	54	9 (17)	
Ki-67			0.3275
−	7	0 (0)	
+	47	12 (26)	
Biomarkers amplification			
KISS1	23		0.4864
Aneuploid	8	1 (13)	
Normal	13	4 (31)	
Amplification	2	1 (50)	
VEGFR	23		0.3401
Aneuploid	1	0 (0)	
Normal	18	6 (33)	
Amplification	4	0 (0)	
Biomarkers protein expression			
KISS1	25		0.6016
Normal	21	7 (33)	
Overexpressed	4	2 (50)	
VEGFR	22		0.6462
Normal	12	4 (33)	
Overexpressed	10	2 (20)	
HIF	38		0.3367
Normal	33	11 (33)	
Overexpressed	5	3 (60)	
eNOS	38		1.000
Normal	34	11 (33)	
Overexpressed	4	1 (25)	
AGTR1	26		*0.0033*
Normal	15	1 (7)	
Overexpressed	11	7 (64)	
Biomarkers gene expression			
VEGF	34		0.3235
Higher	1	1 (100)	
Lower	33	10 (30)	
VEGFR	34		0.0693
Higher	7	0 (0)	
Lower	27	11 (41)	
HIF	34		0.4254
Higher	9	4 (44)	
Lower	25	7 (28)	
eNOS	34		1.000
Higher	1	0 (0)	
Lower	33	11 (33)	
KISS1	34		1.0000
Higher	1	0 (0)	
Lower	33	11 (33)	

Italic values indicate *p*-value <0.05

In the ITT population analysis, a clinical ORR of 89 % (95 % CI 79–95 %) was documented (64/72 included patients), with 42 patients reaching complete response (CR) (58, 95 % CI 46–70 %). Six patients had stable disease (8 %), and 1 patient had disease progression after receiving 2 cycles of AC.

### Study of other potentially predictive factors

Forty-nine patients gave their consent to have the biomarkers analyzed. Table 3 shows pCR rates relative to the explored biological markers in the baseline biopsy, as well as the number of analyzed cases: in some tumor samples, not all biomarkers could be studied due to suboptimal quality of biopsy.

A statistical significant association was observed between Angiotensin II type 1 receptor (AGTR1) protein overexpression and pCR (*p* = 0.0033). No other biomarker significantly correlated with pCR.

Using a non parametric test (Wilcoxon Mann–Whitney) to compare eNOS data expression as continuous variable between patients with pCR and non pCR, an association between higher levels of eNOS and probability of reaching pCR was found (*p* = 0.07 bilateral test, and *p* = 0.03 unilateral test).

**Fig. 2:**
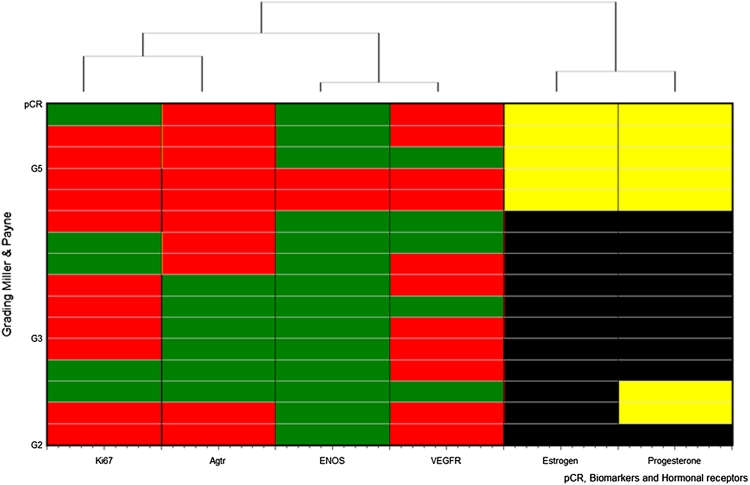
Cluster analysis of relation between anatomopathological response and biomarkers and hormonal receptor status (subset of patients with assessable anatomopathological response and informed biomarkers and hormonal receptors: *N* = 16) *Green* color, expressed biomarker or high proliferative index of Ki67; *red* color, not expressed biomarker or low proliferative index of Ki67; *yellow* color, negative hormone receptor; *black* color, positive hormone receptor. Cluster 1, ER/PgR; Cluster 2, ENOS/VEGFR; Cluster 3, Ki67/AGTR

An analysis cluster was made with those patients with assessable anatomopathological response in whom Ki67, AGTR, ENOS, VGFR, ER, and PgR were evaluated (16 patients). That analysis showed that three clusters (ER/PgR, ENOS/VEGFR, and Ki67/AGTR) explained 80 % of the variability of the anatomopathological response. A graphical representation of that relation is shown in Fig. 2.

## Discussion

HER2 overexpression and HR-status are associated with substantially higher pCR rates [19–25]. In our study, one of the largest published phase II study testing bevacizumab as neoadjuvant treatment of BC, all included patients had HER2-negative disease and 43 (60 %) were HR+: moreover, the median tumor size (physical examination) was high (5 cm). In spite of these unfavorable prognostic features, the reached 24 % pCR rate falls within the range reported for anthracycline and taxane-containing regimens in populations unselected for HER2 status [3, 26–28], as well as of that reported for others with bevacizumab plus chemotherapy [7–9]. It was noteworthy that the 17 % (9/54 patients) pCR rate reached in the subgroup of women with HR+ disease, in line with results of the NSABP B40 study [13], where the effect of adding bevacizumab to neoadjuvant chemotherapy was predominantly in the HR+ subset, although without reach statistic significance. Moreover, recently published data from the GeparQuinto study [14], suggest that the effect of the addition of bevacizumab to EC-docetaxel chemotherapy on pCR rates derived mainly from patients with triple-negative BC: nevertheless, the test for interaction was not significant (*p* = 0.07) in this study, which was not powered to show these differential effects. Randomized studies designed to address the efficacy of bevacizumab in tailored populations are needed to clarify these points.

Regarding clinical responses, our 89 % ORR (ITT population) is among the highest reported in other similar neoadjuvant trials with bevacizumab-based combinations (67–87 % ORR) [6, 8, 9, 11, 29, 30]. Even taking into account that a comparison with previous phase II studies can be only speculative, it was noteworthy that 42 out of 72 patients (58 %) reached a clinical CR, which compares very favorably with that achieved by others [8, 29, 30]. Interestingly, 17/24 non responding patients after AC obtained a further partial or CR to BT. Moreover, 19 out of 33 improved their partial responses to complete responses at the end of BT treatment, suggesting that a significant proportion of clinical and pathological responses may be attributable to the combination of bevacizumab plus docetaxel. These results allowed a remarkable 63 % breast-conserving surgery rate (42/72 patients).

The regimen was very well tolerated, in line with other previously published regimens with bevacizumab as primary treatment of breast cancer [6, 8, 9, 11, 29, 30], with a high proportion of patients (89 %) receiving all treatment as planned. The incidence of grade 3/4 hematological and non-hematological toxicities was low, with only 11/72 patients presenting any AE classified as probably related to any study medication. No toxic death was found and only one patient did not receive bevacizumab for 2 cycles due to uncontrolled hypertension.

This study also evaluated some biological markers as predictors for pCR. In our understanding, this is the first clinical study that suggests that Angiotensin II type 1 receptor (AGTR1) protein overexpression may be related to the pCR to bevacizumab treatment in BC patients. In a similar way, the association between higher levels of eNOS and probability of reaching pCR found in our study should be corroborated in larger studies specifically aimed to address this issue. No statistical correlation between any other studied biomarker and the pathological outcome has been found, probably due to the small number of subjects in each subgroup. In particular, no statistically significant association was found between the proliferation-related levels of Ki67 and the pCR rate, although no pCR was reached in any patient with low Ki67 levels. In a previous study [10], we found a relation between Ki67 positive tumors and pCR; nevertheless, in other similar phase II studies with bevacizumab [8, 11, 12] this correlation did not reach a statistical signification. Further studies on a larger number of patients are therefore required to evaluate the role of these biomarkers.

In conclusion, the high rate of clinical and pathological activity and low incidence of severe toxicity seen with the addition of bevacizumab to this standard sequential schedule of neoadjuvant chemotherapy, even in a population of patients with adverse prognostic factors, suggests that this regimen can be used in randomized trials to address fundamental clinical questions about patient-tailored regimens, timing and duration of bevacizumab therapy. In particular, the identification of reliable biomarkers that predict response or resistance to antiangiogenic treatments and can be used for the selection of patients eligible for therapy with bevacizumab remains a priority. Those strategies would help to individualize more efficiently primary systemic therapy for patients with breast cancer.
